# Supplementary material for: Population-based otoscopic and audiometric assessment of a birth cohort recruited for a pneumococcal vaccine trial 15–18 years earlier: a protocol
Source: BMJ Open. 2021 Feb 17;11(2):e042363. doi: 10.1136/bmjopen-2020-042363 (PMC7893663; doi:10.1136/bmjopen-2020-042363)
Supplement: Author's manuscript [file bmjopen-2020-042363.draft_revisions.pdf]

BMJ Open is committed to open peer review. As part of this commitment we make the peer review history of every article we publish publicly available.

When an article is published we post the peer reviewers' comments and the authors' responses online. We also post the versions of the paper that were used during peer review. These are the versions that the peer review comments apply to.

The versions of the paper that follow are the versions that were submitted during the peer review process. They are not the versions of record or the final published versions. They should not be cited or distributed as the published version of this manuscript.

BMJ Open is an open access journal and the full, final, typeset and author-corrected version of record of the manuscript is available on our site with no access controls, subscription charges or pay-per-view fees (http://bmjopen.bmj.com).

If you have any questions on BMJ Open's open peer review process please email info.bmjopen@bmj.com

BMJ Open

Population based otoscopic and audiometric assessment of a birth cohort recruited for a pneumococcal vaccine trial 15-18 years earlier: Protocol

Journal:	*BMJ Open*
Manuscript ID	bmjopen-2020-042363
Article Type:	Protocol
Date Submitted by the Author:	08-Jul-2020
Complete List of Authors:	Chan, Kenny; University of Colorado Denver School of Medicine, Otolaryngology-Head and Neck Surgery; Children's Hospital Colorado, Pediatric Otolaryngology<br>Carosone-Link, Phyllis; University of Colorado Denver School of Medicine, Pediatrics<br>Bautista, Mary ; Research Institute for Tropical Medicine, Clinical Trials, Epidemiology, and Biostatistics<br>Sanvictores, Diozele ; Research Institute for Tropical Medicine, Clinical Trials, Epidemiology, and Biostatistics<br>Uhler, Kristin; Children's Hospital Colorado, Audiology, Speech-Pathology, and Learning; University of Colorado Denver School of Medicine, Physical Medicine and Rehabilitation<br>Tallo, Veronica; Research Institute for Tropical Medicine, Clinical Trials, Epidemiology and Biostatistics<br>Lucero, Marilla G.; Research Institute for Tropical Medicine, Clinical Trials, Epidemiology and Biostatistics<br>De Jesus, Joanne; Research Institute for Tropical Medicine, Clinical Trials, Epidemiology and Biostatistics<br>Simoes, Eric; Colorado School of Public Health, Epidemiology
Keywords:	Epidemiology < INFECTIOUS DISEASES, Paediatric otolaryngology < OTOLARYNGOLOGY, Audiology < OTOLARYNGOLOGY

SCHOLARONE™
Manuscripts

I, the Submitting Author has the right to grant and does grant on behalf of all authors of the Work (as defined in the below author licence), an exclusive licence and/or a non-exclusive licence for contributions from authors who are: i) UK Crown employees; ii) where BMJ has agreed a CC-BY licence shall apply, and/or iii) in accordance with the terms applicable for US Federal Government officers or employees acting as part of their official duties; on a worldwide, perpetual, irrevocable, royalty-free basis to BMJ Publishing Group Ltd ("BMJ") its licensees and where the relevant Journal is co-owned by BMJ to the co-owners of the Journal, to publish the Work in this journal and any other BMJ products and to exploit all rights, as set out in our licence.

The Submitting Author accepts and understands that any supply made under these terms is made by BMJ to the Submitting Author unless you are acting as an employee on behalf of your employer or a postgraduate student of an affiliated institution which is paying any applicable article publishing charge ("APC") for Open Access articles. Where the Submitting Author wishes to make the Work available on an Open Access basis (and intends to pay the relevant APC), the terms of reuse of such Open Access shall be governed by a Creative Commons licence – details of these licences and which Creative Commons licence will apply to this Work are set out in our licence referred to above.

Other than as permitted in any relevant BMJ Author's Self Archiving Policies, I confirm this Work has not been accepted for publication elsewhere, is not being considered for publication elsewhere and does not duplicate material already published. I confirm all authors consent to publication of this Work and authorise the granting of this licence.

For peer review only

Population based otoscopic and audiometric assessment of a birth cohort recruited for a pneumococcal vaccine trial 15-18 years earlier: Protocol

Kenny H Chan, MD[1,2,3] (Kenny.chan@childrenscolorado.org),

Phyllis Carosone-Link, MS, MSPH[3] (phyllis.carosonelink@cuanschutz.edu),

Mary Thatcher G Bautista, RN, MAN[4] (thatchergonzales@yahoo.com),

Diozele Hazel Sanvictores[4] (diozele_sanvictores@yahoo.com),

Kristin Uhler, PhD[4] (Kristin.Uhler@childrenscolorado.org),

Veronica Tallo, PhD[5] (vickee.popmetrics@gmail.com),

Marilla Lucero, MD[5] (luceromarilla2016@gmail.com),

Joanne De Jesus, MD[5] (dejesus_joanne@yahoo.com),

Eric AF Simões, MBBS, DCH, MD[3,6] (eric.simoes@cuanschutz.edu)

<u>Affiliations</u>

[1]Department of Otolaryngology-Head and Neck Surgery, University of Colorado School of Medicine, Aurora, Colorado, United States

[2] Department of Pediatric Otolaryngology, Children's Hospital Colorado, Aurora, Colorado

For peer review only - http://bmjopen.bmj.com/site/about/guidelines.xhtml

[3]Department of Pediatrics, University of Colorado School of Medicine Aurora Colorado, United States,

[4]Department of Clinical Trials, Epidemiology and Biostatistics, Research Institute for Tropical Medicine Department of Health, Muntinlupa, Manila, Philippines.

[5] Department of Physical Medicine and Rehabilitation, University of Colorado School of Medicine, Department of Audiology, Speech-Pathology, and Learning Children's Hospital Colorado, Aurora, Colorado, United States

[6] Department of Epidemiology Center for Global Health, Colorado School of Public Health, Aurora Colorado, United States

Corresponding Author:

Kenny H Chan, MD

Department of Otolaryngology

Children's Hospital Colorado

13123 E. 16th Ave., Box 455, Aurora Colorado, United States

Tel: 720 777 4776

E-mail: Kenny.Chan@childrenscolorado.org

Manuscript Word Count: 3668; Abstract Word Count: 300

For peer review only

ABSTRACT

Introduction: A cohort of 12,000 children in the Philippines who had enrolled in a 2000-2004 Phase 3 pneumococcal conjugate vaccine for the treatment of radiographically confirmed pneumonia are now being asked to participate in a separate study to assess the cohort's current long-term audiometric and otologic status. This new study would allow assessments of the utility of the pneumococcal vaccine in conferring its protective effects on otitis media (OM) and the long-term sequelae of OM, if any. Lack of trained local healthcare providers in otolaryngology/audiology and testing equipment in Bohol, Philippines necessitates the development of a distinct methodology that would lead to meaningful data analysis.

Methods and Analysis: Reliable data collection and transfer are achieved by a U.S. otolaryngologist/audiologist team training local nurses on all procedures in a didactic and hands-on process. An assortment of portable otolaryngologic and audiologic equipment suitable for field-testing has been acquired including: operating otoscopes (Welch-Allyn^{R}), a video-otoscopes (JedMed^{R}), a tympanometer with distortion product otoacoustic emission (DPOAE) measurements (Path Sentiero^{R}) and a screening audiometer (HearScreen^{R}). Data will then be uploaded to a REDCap database in the U.S.

Tympanometric and audiologic data will be codified through separate conventional algorithms. A team of pediatric otolaryngology advanced practice providers (APPs) have been trained and validated in interpreting video-otoscopy. The protocol for classification of diagnostic outcome variables based on video-otoscopy and tympanometry has been developed and is being utilized by APPs to evaluate all otoscopy data.

For peer review only - http://bmjopen.bmj.com/site/about/guidelines.xhtml

Ethics and Dissemination

Ethics - The study was approved by the Research Institute of Tropical Medicine, Alabang, Manila, Philippines, and the Institution Review Board and the Colorado Multiple Institutional Review Board of the University of Colorado School of Medicine, Aurora, Colorado, USA

Dissemination - Research results will be made available to children and their caregivers with abnormal audiologic outcomes, the funders and other researchers.

For peer review only

STRENGTHS AND LIMITATIONS OF THIS STUDY

- This large-scale study involves data collection in the Philippines and data transfers to the U.S.; thereby, a completely novel population-based audiometric and otoscopic assessment methodology needs to be developed.
- This methodology allows for the efficacy evaluation of an RCT of pneumococcal vaccine conducted between 2000- 2004 on the long-term complications of otitis media (chronic suppurative otitis media and its sequelae on the hearing).
- Despite in-depth training of local nurses to collect data and U.S. APPs to categorize video-otoscopic data, this methodology contains some degree of human factors and subjectivity that may lead to bias.
- This methodology lacks the ability to resolve inferior quality data or missing data due to logistic and geographic constraints resulting in decreased usable data for analysis.

For peer review only

For peer review only - http://bmjopen.bmj.com/site/about/guidelines.xhtml

INTRODUCTION

Otitis media (OM) and its most consequential complication, chronic suppurative otitis media (CSOM), are disproportionally overrepresented in developing countries, particularly in Asia and sub-Saharan Africa.[1] The burden of disease is a spectrum of complications which include hearing loss, tympanic membrane (TM) perforation, otorrhea, adhesive OM/retraction pocket, cholesteatoma and intratemporal and intracranial complications which in turn could have important downstream social, educational and vocational impacts. OM global health initiatives and clinical research in these populations mandate accurate epidemiologic assessments in low- and middle-income countries (LMIC).

However, large-scale otoscopic and audiometric assessment of ear disease in children in LMIC is difficult due to logistical constraints, including a lack of portable equipment and trained audiologists and otolaryngologists with the time and interest for these studies. A survey[2-8] of published reports in the past 15 years with relevance to OM through population studies in LMIC has revealed one or more methodologic deficiencies; namely, small sample size, lacking either otoscopic or audiometric evaluation, and particularly, assessment of CSOM.

Two recent studies have overcome these apparent deficiencies including the documentation of frequencies of CSOM through large-scale population surveys in at-risk regions including Asia (Indonesia)[9] and Africa (Kenya).[10] However, both of these studies relied heavily on onsite local highly-trained otolaryngologists and audiologic personnel. In the Indonesian study, locally trained certified audiologists conducted hearing testing while in the Kenyan study, trained specialized medical officers were utilized to carry out clinical assessments and audiometric

For peer review only - http://bmjopen.bmj.com/site/about/guidelines.xhtml

assessments. Both studies required significant time commitments of local trained audiologic personnel and senior otolaryngologists to conduct the studies. Both studies were carried out by research teams visiting schools and conducting cross-sectional studies over 3 to 5 days at each school that lasted for several months. However, while this strategy works for time limited cross-sectional studies in LMIC, this strategy will not work in long term longitudinal prospective studies.

BACKGROUND

We had to develop a novel methodology to study the impact of an 11-valent pneumococcal conjugate vaccine on long-term audiometric, otologic and cognitive outcomes of a birth cohort of 12,000, previously enrolled in a randomized clinical trial,[11] 15-18 years prior. The study is being conducted on Bohol island Philippines, with limited otolaryngology (2 general otolaryngologists on the island) and audiology (1 facility with a hearing booth) resources. After piloting hands-on training of local nurses in Bohol Philippines, to conduct otoscopic and audiologic assessments,[12] we have refined and standardized all research definitions and protocols outlined in this manuscript.

METHODS & ANALYSIS

A. **Study Site**: Bohol, Philippines is located 640 km south of Manila and its land mass consists of Bohol Island and 75 minor surrounding islands (total area of 4, 821 km^2). It has a population of over 1.3 million inhabitants (2015 census) with a population density of 270/km^2. The local economy is sustained by tourism and agriculture. The 2018 poverty rate (defined as a family of five earning less than PhP 7,337 a month [equivalent to US$144]) in

For peer review only - http://bmjopen.bmj.com/site/about/guidelines.xhtml

Bohol was reported to be 21.1% (http://www.psa.gov.ph/poverty-press-releases/nid/138411).

B. **Subjects of the study**: The study cohort consists of a potential of over 12,000 Filipino children who were enrolled in a pneumococcal vaccine trial conducted in Bohol 2000 to 2004

C. **Study Aim:** The specific aim of the methodology is to obtain otoscopic and audiologic data from enrolled subjects to enable assignment of otitis media related and audiometric diagnostic outcomes to each of the study subjects. To achieve this goal, the methodology has the following key components: 1) portable testing equipment, 2) training of local research personal using the testing equipment, 3) development of a protocol for data collection, storage and transfer, 4) cataloging tympanometric data using published metrics, 5) video-otoscopy data and diagnosis outcome classifications, and 6) training U.S. APPs in video-otoscopy. A detailed description of each of the steps is further elucidated below.

D. Testing equipment

Each testing device was chosen for its portability, technical capabilities, and potential reliable performance in the tropics, particularly, being able to be transported for field use.

1. An otoscope with an operating head (21700, Welch-Allyn[R], Skaneateles Falls, NY, USA) was selected to initially obtain a sufficient view of the TM for subsequent video-otoscopy. Associated micro-instruments were selected to clear cerumen if the TM could not be adequately visualized.

For peer review only - http://bmjopen.bmj.com/site/about/guidelines.xhtml

2. A portable, rechargeable battery-operated video otoscope able to capture and record high-definition (1080p) still images and streaming video was chosen (Horus+ HD Video Otoscope, JedMed[R], St. Louis, MO, USA). Images are initially recorded on SD cards in the field and subsequently transferred to hard discs at the research office.

3. A portable, rechargeable battery-operated tympanometry and distortion product otoacoustic emission (DPOAE) device (Path Sentiero[R], Path Medical, Germering, Germany, model SOD07) was selected. While the device has many functionalities, only a limited number were required for the study. The 226Hz frequency tympanogram issued at a pressure range of -600 to +400 daPa. The tympanometer outputs for each ear include: a tracing viewable by the tester as well as physical measurements of ear canal volume (ml), peak pressure (daPa), peak compliance (ml) and tympanic width (daPa). DPOAE is used as a screening tool and measured at 2, 3, and 4 kHz for each ear. Although detailed results are available through the device, only the basic results with outputs as “pass” or “refer” are to be used since DPOAE is only being used as a screening tool for hearing loss.

4. A portable, rechargeable battery-operated screening audiometry system (HearScreen[R], HearX, Pretoria, South Africa) has been chosen coupled with a smartphone (Samsung Galaxy 12) and an ISO calibrated noise-cancelling headphone (Sennheiser HD280-pro Circumural Headphone [0.5 – 8kHz]). The device uses an automated testing protocol to test pure tone frequencies at 0.5, 1, 2, 4, and 8 kHz for each ear at sound intensity levels of 20 to 70 dB HL. The device then generates a pass or fail interpretation based on published best practice guidelines. Subjects that fail the screening audiogram are

For peer review only - http://bmjopen.bmj.com/site/about/guidelines.xhtml

referred for a diagnostic audiogram at Bohol Hearing Center. All HearX data are stored on remote servers and can be accessed by the research team.

E. Training of local research personnel

The training of local Filipino nurses by a U.S. otolaryngology/audiologist team has been described previously.[12] Briefly, 4 nurses were trained didactically on the topic of OM and the basics of audiology and its relationship to OM clinically. Then they were given hands-on training in otoscopy, cerumen removal, video-otoscopy, and all of the audiologic equipment to perform tympanometry, DPOAE, and screening audiograms. The training session culminated in the nursing team obtaining video-otoscopic, audiologic, and clinical data from 47 subjects under direct supervision of the U.S. otolaryngologist/audiologist team.

F. Protocols for data collection, storage and transfer protocols

Research data collection by the local research team include: 1) case report forms; 2) video-otoscopy and audiologic testing; and 3) miscellaneous data.

1. Case report form (CRF)

Each research subject has an original paper CRF which contains patient identifiers and clinical information entered by the research personnel following a parental interview with ear-specific past medical history (i.e. ear infection, discharge, TM perforation, and hearing loss). CRFs collected during the week are uploaded by the research nurse into a REDCap database hosted at the University of Colorado Denver School of Medicine,[26] Aurora, Colorado, USA.

For peer review only - http://bmjopen.bmj.com/site/about/guidelines.xhtml

2. Video-otoscopy and audiologic testing

Subjects whose TMs are devoid of cerumen or whose cerumen can be cleaned by the research nurses undergo video-otoscopy and audiologic testing. All subjects whose cerumen cannot be cleaned adequately are given a bottle of mineral oil and a medicine dropper and advised to use 5 drops twice a day for 7 days and asked to return for another assessment. However, subjects that fail to have adequate cerumen removal after the subsequent visit or had active otorrhea are referred to the local otolaryngologist for further management

The research nurses record the 2 ears of a research subject in a regimented sequence for ease of review by the APPs at a subsequent time (right ear still photo, left ear still photo, right ear video, and left ear video). SD cards are downloaded at the end of each weekday and images stored on 2 hard disks (original and backup disks). Data is transferred to the US by external drives

Audiologic testing data are processed in the following manner. The 4 discreet outputs produced by the tympanometer are used to determine tympanogram types. An overlay “rectangle” onto the tympanometer tracing is used by the manufacturer to include most “normal” tympanograms within these confines as defined by Jerger[13] to assist with diagnosis. The local research team uses this real-time judgement call for a “pass” or “fail” depending if the peak of the tracing is within the “normal” ranges as dictated by the “rectangle.” DPOAE results are also automatically determined by the device as either “pass” or “refer.” Hearing screening done on subjects using the HearScreen

For peer review only - http://bmjopen.bmj.com/site/about/guidelines.xhtml

device also classifies subjects as "pass" or "fail" by the device. All subjects who fail the tympanogram, DPOAE, or HearScreen are referred to Bohol Hearing Center for diagnostic audiometry. All raw tympanogram, automated DPOAE, automated HearScreen, and raw HearX data are entered by the research supervisor to the REDCap database on a weekly basis.

3. Miscellaneous data

All audiologic data from Bohol Hearing Center, as well as all otologic diagnoses made by the island otolaryngologist, are also entered into the REDCap database by the research supervisor on an as-needed basis.

G. Cataloging of tympanometric data

The tympanometric data for each ear consisting of ear canal volume (ml), peak pressure (daPa), peak compliance (ml), and tympanic width (daPa) are sorted based on the classic classification established by Jerger: A, B, C, A_s, and A_d types. As such, a portion of data would result in tympanometric parameters landing outside of the confines for each type defined by Jerger.[13] Since the research team viewed it imperative that each video-otoscopy be accompanied by a tympanogram type to aid in video-otoscopy interpretation, an *ad hoc* methodology had to be developed to capture the "non-classifiable" tympanograms. The study otolaryngologist and a senior audiologist independently will read all "non-classifiable" tympanograms, and discordant classifications will be adjudicated by a second senior audiologist. The majority vote will confirm the classification type.

For peer review only - http://bmjopen.bmj.com/site/about/guidelines.xhtml

H. Video-otoscopy data and diagnosis outcome classifications

Video-otoscopy analysis leading to disease classification takes into account all otoscopic abnormalities associated with acute OM, chronic non-suppurative OM, and CSOM, as well as a non-OM diagnoses (e.g. otitis externa) summarized in Table 1. APPs document findings rather than immediately arrive at a diagnosis. Each video-otoscopic image is associated with a tympanogram type as to simulate pneumatic otoscopy, a proxy for the gold standard to diagnose middle-ear effusion.[14,15] The gold standard for the presence of middle-ear fluid is detected only by myringotomy ;however, this being impossible, the proxy methodology used here is based on the decisions made by the senior pediatric otolaryngologist with over 30 years of performing otologic procedures for complications, from OM including tympanoplasties and mastoidectomies. Given it would be impractical and impossible for the pediatric otolaryngologist to view thousands of video-otoscopies, experienced otoscopists (APPs) have been trained and validated in identifying otoscopic abnormalities listed in Table 1.

All video-otoscopies that are deemed as poor quality by the APP are reviewed by the otolaryngologist of the study and would remained classified as poor quality or into one or more of the following TM findings.

Video-otoscopic findings for each ear will then merged with the 4 tympanometric output parameters and clinical past medical history to arrive at discreet diagnoses (Figure 1). Four broad diagnostic categories with definitions include normal, mild, moderate, and severe ear disease. If two ears of a subject have disparate levels of middle-ear disease, the subject would be classified as having the more serious form of ear disease for the purpose of analysis.

For peer review only - http://bmjopen.bmj.com/site/about/guidelines.xhtml

1. Normal

No evidence of ear disease as determined by no disease-related video-otoscopic findings regardless of tympanometric abnormal states and clinical past medical history of ear disease.

2. Mild Ear Disease

- Acute otitis media (AOM) – The presence of at least two of the following three otoscopic signs: 1) white, yellow, amber, or blue TM (i.e., abnormal color), 2) opacification not due to scarring, 3) tympanometry peak compliance <0.2 ml or tympanometric width > 200 daPa on tympanometry; or bubbles or air-fluid interfaces; and one of the following three signs/symptoms: a) ear pain, b) redness of the TM, c) fullness or bulging of the TM or acute purulent otorrhea not due to otitis externa.[16-18]

- Otitis media with effusion (OME) – The presence of at least two of the following three otoscopic signs: 1) white, yellow, amber, or blue TM (i.e., abnormal color), 2) opacification not due to scarring, 3) tympanometry peak compliance <0.2 ml or tympanometric width > 200 daPa from tympanogram; or bubbles or air-fluid interfaces; and the absence of the following four signs/symptoms: a) ear pain, b) redness of the TM, c) fullness or bulging of the TM, or d) acute purulent otorrhea not due to otitis externa.[16-18]

- Healed Perforation – Otherwise normal TM with a thinned-out area of TM.

For peer review only - http://bmjopen.bmj.com/site/about/guidelines.xhtml

- Myringosclerosis – a hardening of the ear drum as calcium deposits form on the ear drum and middle ear; peak compliance on tympanogram > 5.0 ml.

3. Moderate Ear Disease

- Dry Perforation – perforation of the TM without otorrhea or ear canal volume ≥2 ml on tympanogram.

- Adhesive otitis media – Intact TM but retraction or retraction pocket seen.

4. Severe Ear Disease

- Chronic Suppurative Otitis Media (CSOM), active – perforation of the TM with otorrhea duration of more than 2 weeks; or TM with retraction pocket unable to see deepest part, with granulation tissue, squamous debris, or otorrhea.

I. **Protocol to deal with ears with incomplete data set**

It is expected some subjects or ears would not have a complete data set. Reasons would include poor quality collection techniques, uploading error and the subjects not following through with seeing the island otolaryngologist or completing the audiogram at Bohol Hearing Center.

When the case report form indicates otorrhea and no other data is available/discernable, these ears are classified as CSOM. When the case report form indicates no otorrhea or ear pain and only the tympanogram is available, the ears are classified either as normal or OME based on type A or types B/C, respectively. In ears without tympanograms, ear disease classification is solely based on video-otoscopy. When the case report form notes no ear pain; tympanogram is

For peer review only

For peer review only - http://bmjopen.bmj.com/site/about/guidelines.xhtml

missing and video-otoscopy is missing/undiscernible, these ears are deemed “missing data” and will not be included as part of the final analysis.

J. Training of U.S. advanced practice providers

Three outpatient APPs from the Department of Otolaryngology at Children’s Hospital Colorado with varying length of tenure and training in otoscopy (≥7 years of clinical experience) were trained in reading video-otoscopic images using didactic in-person training methods to identify the 14 possible otoscopic signs described in Table 1.

K. Patient and Public Involvement

No patient and public involvement took place in the design, or conduct, or reporting, or dissemination plans of the research.

DISCUSSION

The methodology discussed in this manuscript will be utilized to analyze a potential cohort of 12,000 children previously enrolled in a pneumococcal vaccine trial in Bohol, Philippines and will now be assessed 12 to 18 years later for the vaccine’s effects on otoscopic and audiologic endpoints. It is unique in several aspects: size of the cohort, clinical endpoints, and complexity of data analysis.

The sample sizes of previously published randomized controlled pneumococcal conjugate vaccine trials have been clearly summarized by a recently published Cochrane Review.[19] Our cohort of over 12,000 children to be assessed likely would be lowered since attrition might occur due to inability to locate the families due to relocation or immigration after 15 to 18

For peer review only

years, as well as parental refusal to participate. Regardless, the biggest pneumococcal vaccine trials were published by Black et al in 2000[20-22] and Tregmaghi et al in 2014[23] involving 37,868 and 24,000 subjects, respectively. Black's study was conducted in the U.S., and acute OM was the only ear-related outcome.[21] Tregmaghi study was done in Central and South American countries, but only 7,214 children from Panama were included in the OM portion of the study.[23] Prymula et al in 2006 published an acute OM trial using an 11-valent pneumococcal vaccine conjugated to *Hemophilus influenzae*-derived protein D trial in 4,968 subjects.[24] This trial was conducted in the Czech Republic and Slovakia, and tympanocentesis and pneumococcal subtyping were done. A recent open label trial of maternal immunization with a 23 valent polysaccharide pneumococcal vaccine in 227 Indigenous mothers in the Northern Territories, Australia, showed no impact on OM related infant outcomes at 7 months of age.[25]

The clinical outcomes for the randomized controlled trials have included the reduction of pneumococcal OM, recurrent OM, OM visits, and tympanostomy tube insertions.[20-24,30] None of these randomized controlled trials were focused on the long-term sequalae of OM in terms of changes in the TM as evaluated by otoscopy and changes in hearing as evaluated by audiometry. While prevention of disease is compelling for the use of the pneumococcal vaccine, preventing of long-term sequalae are just as important. Trials conducted in the Indigenous populations in Australia that examined long-term sequelae occurred after licensure of pneumococcal vaccines in Australia and so followed before/after designs.[26-29] Thus, our study will be among the largest randomized controlled pneumococcal vaccine trials for OM and the only one to study the long-term effects on OM sequelae 15 - 18 years after the initial trial.

For peer review only - http://bmjopen.bmj.com/site/about/guidelines.xhtml

The complexity of the methodology dealing with data lies at several levels. First, it requires the raw data collected by the local research team be uniform and robust. Despite a local research supervisor ensuring good quality data were recorded and uploaded, a certain degree of data scrubbing will be required on the U.S. side. Tympanometric and audiologic data are codified based on published definitions of classifications. An algorithm has also been developed to derive OM diagnostic endpoints from all video-otoscopic images that was reliant on the raw images and tympanogram types.

CONCLUSIONS

The overall construct of the methodology may serve as a platform for conducting large scale OM research in LMIC, particularly, if the objectives include garnering otoscopic and audiologic determinates and assessment of chronic and long-term OM complications.

ETHICS AND DISSEMINATION

A. Ethics

The "Do No Harm" precept has been inculcated in the study. Despite the healthcare discrepancy that exists between the U.S. and Bohol, Philippines, accommodations have been made in the methodology to refer the research subjects to local healthcare professionals. The local field workers will be instructed to refer subjects to Bohol Hearing Center for failed OAEs, tympanometry, and/or screening audiometry and to the local otolaryngologist for ear pathology (inability to remove cerumen after instillation of mineral oil from a previous visit, otorrhea and suspicion for cholesteatoma).

For peer review only - http://bmjopen.bmj.com/site/about/guidelines.xhtml
For peer review only

The study was approved by the Research Institute of Tropical Medicine, Alabang, Manila, Philippines, and the Institution Review Board and the Colorado Multiple Institutional Review Board of the University of Colorado School of Medicine, Aurora, Colorado, USA.

Study data are collected and managed using REDCap electronic data capture tools hosted at University of Colorado. REDCap (Research Electronic Data Capture) is a secure, web-based application designed to support data capture for research studies, providing: 1) an intuitive interface for validated data entry; 2) audit trails for tracking data manipulation and export procedures; 3) automated export procedures for seamless data downloads to common statistical packages; and 4) procedures for importing data from external sources. Data are stored, managed and processed within the system. Data are exported as SAS files to secure servers on the university of Colorado and Children's Hospital Colorado systems, where data will be analyzed.

B. Dissemination Research data is privately held. Research results will be made available to children and their caregivers with abnormal audiologic outcomes, the funders and other researchers. After publication anonymized and curated data, will be made available to researchers following guidelines of the Bill and Melinda Gates foundation. Data will be published in open access journals only.

For peer review only

For peer review only - http://bmjopen.bmj.com/site/about/guidelines.xhtml

Authors' Contributions

Conception and acquisition of funding- Simoes

Epidemiologic and Field Design – Simoes, Tallo, Lucero, De Jesus, Bautista, Sanvictores

Otoralyngologic Study design: Chan, Uhler, Simoes, Carosone-Link, Uhler

Audiologic study design: Uhler, Chan, Simoes, Carosone-Link, Uhler

Analysis – Simoes, Chan, Carosone-Link, Uhler

Redcap Database creation – Carosone-Link

Drafting and revision – all authors

For peer review only

For peer review only - http://bmjopen.bmj.com/site/about/guidelines.xhtml

Funding Statement

This study was supported by the Bill & Melinda Gates Foundation (grant number OPP1142570) and Colorado CTSA Grants UL1TR002535, KL2TR002534, and TL1TR002533 and UL1 RR025780. The contents of this report are solely the responsibility of the authors and do not necessarily represent the official views of their institutions or organizations or of the sponsors. The funders did not participate in any aspect of the study, including study-conduct, data collection, analyses of the data or the write-up of the manuscript. The corresponding authors confirm that they had full access to all the data in the study and had final responsibility for the decision to submit for publication.

Competing interests

The authors declare that they have no competing interests.

Acknowledgements

The authors, on behalf of study team, would like to thank the other nurses who were the subjects of training: Nino Carlo Salutan, Leslie Salutan, Marelie Dagupan and Shobelle Anunciado; the staff of Research Institute of Tropical Medicine, Manila Philippines for their dedication to their patients, including our trial participants; Deborah Hayes, PhD who was instrumental in developing the audiology protocols.

For peer review only - http://bmjopen.bmj.com/site/about/guidelines.xhtml

REFERENCES:

1. Acuin J. Chronic suppurative otitis media : burden of illness and management options. Geneva: World Health Organization; 2004.
2. Adebola SO, Ayodele SO, Oyelakin OA, Babarinde JA, Adebola OE. Pre-school hearing screening: profile of children from Ogbomoso, Nigeria. *Int J Pediatr Otorhinolaryngol* 2013; **77**(12): 1987-91.
3. Adhikari P, Joshi S, Baral D, Kharel B. Chronic suppurative otitis media in urban private school children of Nepal. *Braz J Otorhinolaryngol* 2009; **75**(5): 669-72.
4. Feniman MR, Souza AG, Jorge JC, Lauris JR. Otoscopic and tympanometric findings in infants with cleft lip and palate. *Braz J Otorhinolaryngol* 2008; **74**(2): 248-52.
5. Mahomed-Asmail F, Swanepoel de W, Eikelboom RH. Hearing loss in urban South African school children (grade 1 to 3). *Int J Pediatr Otorhinolaryngol* 2016; **84**: 27-31.
6. Okur E, Yildirim I, Akif Kilic M, Guzelsoy S. Prevalence of otitis media with effusion among primary school children in Kahramanmaras, in Turkey. *Int J Pediatr Otorhinolaryngol* 2004; **68**(5): 557-62.
7. Smith AF, Ianacone DC, Ensink RJH, Melaku A, Casselbrant ML, Isaacson G. Prevalence of hearing-loss among HAART-treated children in the Horn of Africa. *Int J Pediatr Otorhinolaryngol* 2017; **98**: 166-70.
8. Zakzouk SM, AbdulJawad KA. Point prevalence of type B tympanogram in children. *Saudi Med J* 2002; **23**(6): 708-10.
9. Anggraeni R, Hartanto WW, Djelantik B, et al. Otitis media in indonesian urban and rural school children. *Pediatr Infect Dis J* 2014; **33**(10): 1010-5.
10. Simoes EA, Kiio F, Carosone-Link PJ, Ndegwa SN, Ayugi J, Macharia IM. Otitis Media and Its Sequelae in Kenyan Schoolchildren. *Journal of the Pediatric Infectious Diseases Society* 2015.
11. Lucero MG, Nohynek H, Williams G, et al. Efficacy of an 11-valent pneumococcal conjugate vaccine against radiologically confirmed pneumonia among children less than 2 years of age in the Philippines: a randomized, double-blind, placebo-controlled trial. *Pediatr Infect Dis J* 2009; **28**(6): 455-62.
12. Chan KH, Dreith S, Uhler KM, et al. Large-scale otoscopic and audiometric population assessment: A pilot study. *Int J Pediatr Otorhinolaryngol* 2018; **117**: 148-52.
13. Jerger J. Clinical experience with impedance audiometry. *Arch Otolaryngol* 1970; **92**(4): 311-24.
14. Nozza RJ, Bluestone CD, Kardatzke D, Bachman R. Identification of middle ear effusion by aural acoustic admittance and otoscopy. *Ear Hear* 1994; **15**(4): 310-23.
15. Kaleida PH, Fireman P. Diagnostic assessment of otitis media. *Clin Allergy Immunol* 2000; **15**: 247-62.
16. Diagnosis and management of acute otitis media. *Pediatrics* 2004; **113**(5): 1451-65.
17. Morris PS, Leach AJ, Halpin S, et al. An overview of acute otitis media in Australian Aboriginal children living in remote communities. *Vaccine* 2007; **25**(13): 2389-93.
18. Pellman H. All otitis media is not created equal. *J Pediatr* 1999; **135**(2 Pt 1): 264.

For peer review only

19. Fortanier AC, Venekamp RP, Boonacker CW, et al. Pneumococcal conjugate vaccines for preventing acute otitis media in children. *The Cochrane database of systematic reviews* 2019; **5**: Cd001480.
20. Black SB, Shinefield HR, Ling S, et al. Effectiveness of heptavalent pneumococcal conjugate vaccine in children younger than five years of age for prevention of pneumonia. *Pediatr Infect Dis J* 2002; **21**(9): 810-5.
21. Fireman B, Black SB, Shinefield HR, Lee J, Lewis E, Ray P. Impact of the pneumococcal conjugate vaccine on otitis media. *Pediatr Infect Dis J* 2003; **22**(1): 10-6.
22. Black S, France EK, Isaacman D, et al. Surveillance for invasive pneumococcal disease during 2000-2005 in a population of children who received 7-valent pneumococcal conjugate vaccine. *Pediatr Infect Dis J* 2007; **26**(9): 771-7.
23. Tregnaghi MW, Saez-Llorens X, Lopez P, et al. Efficacy of pneumococcal nontypable Haemophilus influenzae protein D conjugate vaccine (PHiD-CV) in young Latin American children: A double-blind randomized controlled trial. *PLoS Med* 2014; **11**(6): e1001657.
24. Prymula R, Peeters P, Chrobok V, et al. Pneumococcal capsular polysaccharides conjugated to protein D for prevention of acute otitis media caused by both Streptococcus pneumoniae and non-typable Haemophilus influenzae: a randomised double-blind efficacy study. *Lancet* 2006; **367**(9512): 740-8.
25. Binks MJ, Moberley SA, Balloch A, et al. PneuMum: Impact from a randomised controlled trial of maternal 23-valent pneumococcal polysaccharide vaccination on middle ear disease amongst Indigenous infants, Northern Territory, Australia. *Vaccine* 2015; **33**(48): 6579-87.
26. Mackenzie GA, Carapetis JR, Leach AJ, Morris PS. Pneumococcal vaccination and otitis media in Australian Aboriginal infants: comparison of two birth cohorts before and after introduction of vaccination. *BMC Pediatr* 2009; **9**: 14.
27. Leach AJ, Wigger C, Andrews R, Chatfield M, Smith-Vaughan H, Morris PS. Otitis media in children vaccinated during consecutive 7-valent or 10-valent pneumococcal conjugate vaccination schedules. *BMC Pediatr* 2014; **14**: 200.
28. Leach AJ, Mulholland EK, Santosham M, et al. Pneumococcal conjugate vaccines PREVenar13 and SynflorIX in sequence or alone in high-risk Indigenous infants (PREV-IX_COMBO): protocol of a randomised controlled trial. *BMJ open* 2015; **5**(1): e007247.
29. Leach AJ, Wigger C, Beissbarth J, et al. General health, otitis media, nasopharyngeal carriage and middle ear microbiology in Northern Territory Aboriginal children vaccinated during consecutive periods of 10-valent or 13-valent pneumococcal conjugate vaccines. *Int J Pediatr Otorhinolaryngol* 2016; **86**: 224-32.
30. Pettigrew MM, Alderson MR, Bakaletz LO, et al. Panel 6: Vaccines. *Otolaryngol Head Neck Surg* 2017; **156**(4_suppl): S76-s87.

For peer review only

Table 1. List of video-otoscopic finds depicting physical findings associated with otitis media

- Redness of TM
- Opacification not due to scarring (dullness; thickened; webbing)
- Bubbles or air-fluid interfaces or fluid
- Abnormal TM color: white, yellow, amber, or blue
- Fullness or bulging of TM
- Thinned out area of TM or healed perforation seen in 360°
- Tympanosclerosis/Scarring
- Perforation SMALL (<25% or TM)
- Perforation LARGE (≥25% or TM)
- Retraction/Retraction pocket/generally thinned out but not healed TM
- Retraction/ pocket bottom not seen
- Otorrhea not due to otitis externa (moisture)
- Granulation tissue or squamous debris
- Otitis externa

For peer review only

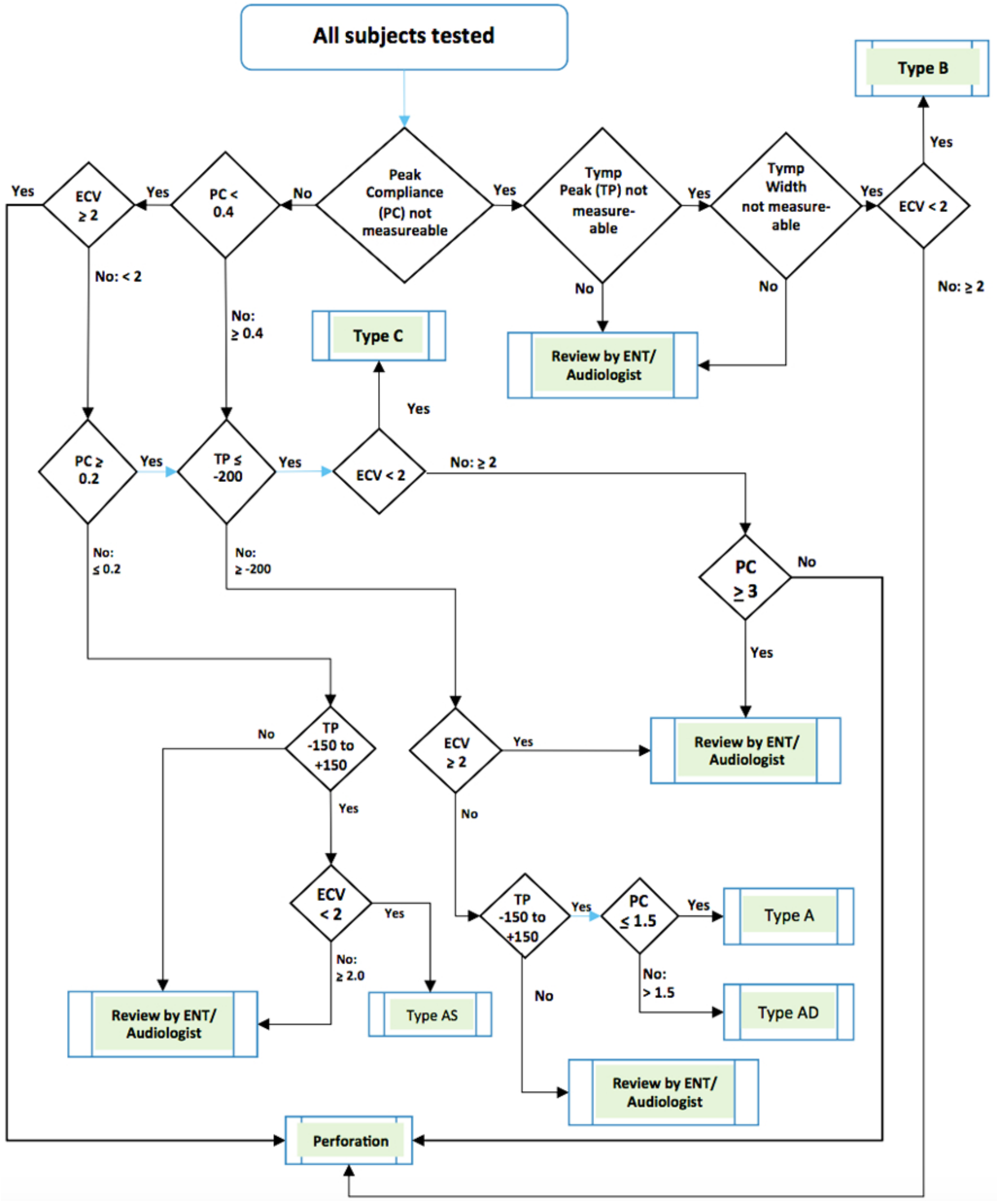

Figure 1. Schema in classifying tympanometric types based on tympanometer outputs (accordance with Jerger classification)

74x90mm (300 x 300 DPI)

BMJ Open

Population based otoscopic and audiometric assessment of a birth cohort recruited for a pneumococcal vaccine trial 15-18 years earlier: Protocol

Journal:	*BMJ Open*
Manuscript ID	bmjopen-2020-042363.R1
Article Type:	Protocol
Date Submitted by the Author:	11-Jan-2021

Complete List of Authors:	Chan, Kenny; University of Colorado Denver School of Medicine, Otolaryngology-Head and Neck Surgery; Children's Hospital Colorado, Pediatric Otolaryngology<br>Carosone-Link, Phyllis; University of Colorado Denver School of Medicine, Pediatrics<br>Bautista, Mary ; Research Institute for Tropical Medicine, Clinical Trials, Epidemiology, and Biostatistics<br>Sanvictores, Diozele ; Research Institute for Tropical Medicine, Clinical Trials, Epidemiology, and Biostatistics<br>Uhler, Kristin; Children's Hospital Colorado, Audiology, Speech-Pathology, and Learning; University of Colorado Denver School of Medicine, Physical Medicine and Rehabilitation<br>Tallo, Veronica; Research Institute for Tropical Medicine, Clinical Trials, Epidemiology and Biostatistics<br>Lucero, Marilla G.; Research Institute for Tropical Medicine, Clinical Trials, Epidemiology and Biostatistics<br>De Jesus, Joanne; Research Institute for Tropical Medicine, Clinical Trials, Epidemiology and Biostatistics<br>Simoes, Eric; Colorado School of Public Health, Epidemiology

**Primary Subject Heading**:	Ear, nose and throat/otolaryngology
Secondary Subject Heading:	Epidemiology
Keywords:	Epidemiology < INFECTIOUS DISEASES, Paediatric otolaryngology < OTOLARYNGOLOGY, Audiology < OTOLARYNGOLOGY

SCHOLARONE™ Manuscripts

I, the Submitting Author has the right to grant and does grant on behalf of all authors of the Work (as defined in the below author licence), an exclusive licence and/or a non-exclusive licence for contributions from authors who are: i) UK Crown employees; ii) where BMJ has agreed a CC-BY licence shall apply, and/or iii) in accordance with the terms applicable for US Federal Government officers or employees acting as part of their official duties; on a worldwide, perpetual, irrevocable, royalty-free basis to BMJ Publishing Group Ltd ("BMJ") its licensees and where the relevant Journal is co-owned by BMJ to the co-owners of the Journal, to publish the Work in this journal and any other BMJ products and to exploit all rights, as set out in our licence.

The Submitting Author accepts and understands that any supply made under these terms is made by BMJ to the Submitting Author unless you are acting as an employee on behalf of your employer or a postgraduate student of an affiliated institution which is paying any applicable article publishing charge ("APC") for Open Access articles. Where the Submitting Author wishes to make the Work available on an Open Access basis (and intends to pay the relevant APC), the terms of reuse of such Open Access shall be governed by a Creative Commons licence – details of these licences and which Creative Commons licence will apply to this Work are set out in our licence referred to above.

Other than as permitted in any relevant BMJ Author's Self Archiving Policies, I confirm this Work has not been accepted for publication elsewhere, is not being considered for publication elsewhere and does not duplicate material already published. I confirm all authors consent to publication of this Work and authorise the granting of this licence.

For peer review only

Population based otoscopic and audiometric assessment of a birth cohort recruited for a pneumococcal vaccine trial 15-18 years earlier: Protocol

Kenny H Chan, MD[1,2,3] (Kenny.chan@childrenscolorado.org),

Phyllis Carosone-Link, MS, MSPH[3] (phyllis.carosonelink@cuanschutz.edu),

Mary Thatcher G Bautista, RN, MAN[4] (thatchergonzales@yahoo.com),

Diozele Hazel Sanvictores[4] (diozele_sanvictores@yahoo.com),

Kristin Uhler, PhD[4] (Kristin.Uhler@childrenscolorado.org),

Veronica Tallo, PhD[5] (vickee.popmetrics@gmail.com),

Marilla Lucero, MD[5] (luceromarilla2016@gmail.com),

Joanne De Jesus, MD[5] (dejesus_joanne@yahoo.com),

Eric AF Simões, MBBS, DCH, MD[3,6] (eric.simoes@cuanschutz.edu)

Affiliations

[1]Department of Otolaryngology-Head and Neck Surgery, University of Colorado School of Medicine, Aurora, Colorado, United States

[2] Department of Pediatric Otolaryngology, Children's Hospital Colorado, Aurora, Colorado

For peer review only

[3]Department of Pediatrics, University of Colorado School of Medicine Aurora Colorado, United States,

[4]Department of Clinical Trials, Epidemiology and Biostatistics, Research Institute for Tropical Medicine Department of Health, Muntinlupa, Manila, Philippines.

[5] Department of Physical Medicine and Rehabilitation, University of Colorado School of Medicine, Department of Audiology, Speech-Pathology, and Learning Children's Hospital Colorado, Aurora, Colorado, United States

[6] Department of Epidemiology Center for Global Health, Colorado School of Public Health, Aurora Colorado, United States

Corresponding Author:

Kenny H Chan, MD

Department of Otolaryngology

Children's Hospital Colorado

13123 E. 16th Ave., Box 455, Aurora Colorado, United States

Tel: 720 777 4776

E-mail: Kenny.Chan@childrenscolorado.org

Manuscript Word Count: 3668; Abstract Word Count: 300

For peer review only

ABSTRACT

Introduction: A cohort of 12,000 children in the Philippines who had enrolled in a 2000-2004 Phase 3 11-valent pneumococcal conjugate vaccine for the prevention of radiographically confirmed pneumonia are now being asked to participate in a separate study to assess the cohort's current long-term audiometric and otologic status. This new study would allow assessments of the utility of the pneumococcal vaccine in conferring its protective effects on the long-term sequelae of otitis media (OM), if any. Lack of trained local healthcare providers in otolaryngology/audiology and testing equipment in Bohol, Philippines necessitates the development of a distinct methodology that would lead to meaningful data analysis.

Methods and Analysis: Reliable data collection and transfer are achieved by a U.S. otolaryngologist/audiologist team training local nurses on all procedures in a didactic and hands-on process. An assortment of portable otolaryngologic and audiologic equipment suitable for field-testing has been acquired including: an operating otoscope (Welch-Allyn^R), a video-otoscope (JedMed^R), a tympanometer with distortion product otoacoustic emission (DPOAE) measurements (Path Sentiero^R) and a screening audiometer (HearScreen^R). Data will then be uploaded to a REDCap database in the U.S.

Tympanometric and audiologic data will be codified through separate conventional algorithms. A team of pediatric otolaryngology advanced practice providers (APPs) have been trained and validated in interpreting video-otoscopy. The protocol for classification of diagnostic outcome variables based on video-otoscopy and tympanometry has been developed and is being utilized by APPs to evaluate all otoscopy data.

For peer review only

Ethics and Dissemination

Ethics - The study was approved by the Research Institute of Tropical Medicine, Alabang, Manila, Philippines, and the Institution Review Board and the Colorado Multiple Institutional Review Board of the University of Colorado School of Medicine, Aurora, Colorado, USA

Dissemination - Research results will be made available to children and their caregivers with abnormal audiologic outcomes, the funders and other researchers.

For peer review only

For peer review only - http://bmjopen.bmj.com/site/about/guidelines.xhtml

STRENGTHS AND LIMITATIONS OF THIS STUDY

- This large-scale study involves data collection in the Philippines and data transfers to the U.S.; thereby, a completely novel population-based audiometric and otoscopic assessment methodology needs to be developed.
- This methodology allows for the efficacy evaluation of an RCT of pneumococcal vaccine conducted between 2000- 2004 on the long-term complications of otitis media (chronic suppurative otitis media and its sequelae on the hearing).
- Despite in-depth training of local nurses to collect data and U.S. APPs to categorize video-otoscopic data, this methodology contains some degree of human factors and subjectivity that may lead to bias.
- This methodology lacks the ability to resolve inferior quality data or missing data due to logistic and geographic constraints resulting in decreased usable data for analysis.

For peer review only

INTRODUCTION

Otitis media (OM) and its most consequential complication, chronic suppurative otitis media (CSOM), are disproportionally overrepresented in developing countries, particularly in Asia and sub-Saharan Africa.[1] The burden of disease is a spectrum of complications which include hearing loss, tympanic membrane (TM) perforation, otorrhea, adhesive OM/retraction pocket, cholesteatoma and intratemporal and intracranial complications which in turn could have important downstream social, educational and vocational impacts. OM global health initiatives and clinical research in these populations mandate accurate epidemiologic assessments in low- and middle-income countries (LMIC).

However, large-scale otoscopic and audiometric assessment of ear disease in children in LMIC is difficult due to logistical constraints, including a lack of portable equipment and trained audiologists and otolaryngologists with the time and interest for these studies. A survey[2-8] of published reports in the past 15 years with relevance to OM through population studies in LMIC has revealed one or more methodologic deficiencies; namely, small sample size, lacking either otoscopic or audiometric evaluation, and particularly, assessment of CSOM.

Two recent studies have overcome these apparent deficiencies including the documentation of frequencies of CSOM through large-scale population surveys in at-risk regions including Asia (Indonesia)[9] and Africa (Kenya).[10] However, both of these studies relied heavily on onsite local highly-trained otolaryngologists and audiologic personnel. In the Indonesian study, locally trained certified audiologists conducted hearing testing while in the Kenyan study, trained specialized medical officers were utilized to carry out clinical assessments and audiometric

For peer review only - http://bmjopen.bmj.com/site/about/guidelines.xhtml

assessments. Both studies required significant time commitments of local trained audiologic personnel and senior otolaryngologists to conduct the studies. Both studies were carried out by research teams visiting schools and conducting cross-sectional studies over 3 to 5 days at each school that lasted for several months. However, while this strategy works for time limited cross-sectional studies in LMIC, this strategy will not work in long term longitudinal prospective studies.

BACKGROUND

We had to develop a novel methodology to study the impact of an 11-valent pneumococcal conjugate vaccine on long-term audiometric and otologic outcomes of a birth cohort of 12,000, previously enrolled in a randomized clinical trial,[11] 15-18 years prior. The study is being conducted on Bohol island Philippines, with limited otolaryngology (2 general otolaryngologists on the island) and audiology (1 facility with a hearing booth) resources. After piloting hands-on training of local nurses in Bohol Philippines, to conduct otoscopic and audiologic assessments,[12] we have refined and standardized all research definitions and protocols outlined in this manuscript.

METHODS & ANALYSIS

A. **Study Site**: Bohol, Philippines is located 640 km south of Manila and its land mass consists of Bohol Island and 75 minor surrounding islands (total area of 4, 821 km^2). It has a population of over 1.3 million inhabitants (2015 census) with a population density of 270/km^2. The local economy is sustained by tourism and agriculture. The 2018 poverty rate (defined as a family of five earning less than PhP 7,337 a month [equivalent to US$144]) in

For peer review only

Bohol was reported to be 21.1% (http://www.psa.gov.ph/poverty-press-releases/nid/138411).

B. **Subjects of the study**: The study cohort consists of a potential of over 12,000 Filipino children who were enrolled in a pneumococcal vaccine trial conducted in Bohol 2000 to 2004 between the ages of 6 to 10 weeks. For the current study, these same subjects will be approximately between 16 to 20 years of age.

C. **Study Aim:** The specific aim of the methodology is to obtain otoscopic and audiologic data from enrolled subjects to enable assignment of otitis media related and audiometric diagnostic outcomes to each of the study subjects. To achieve this goal, the methodology has the following key components: 1) portable testing equipment, 2) training of local research personal using the testing equipment, 3) development of a protocol for data collection, storage and transfer, 4) cataloging tympanometric data using published metrics, 5) video-otoscopy data and diagnosis outcome classifications, and 6) training U.S. APPs in video-otoscopy. A detailed description of each of the steps is further elucidated below.

D. Testing equipment

Each testing device was chosen for its portability, technical capabilities, and potential reliable performance in the tropics, particularly, being able to be transported for field use.

1. An otoscope with an operating head (21700, Welch-Allyn^{R}, Skaneateles Falls, NY, USA) was selected to initially obtain a sufficient view of the TM for subsequent video-otoscopy. Associated micro-instruments were selected to clear cerumen if the TM could not be adequately visualized.

For peer review only - http://bmjopen.bmj.com/site/about/guidelines.xhtml

2. A portable, rechargeable battery-operated video otoscope able to capture and record high-definition (1080p) still images and streaming video was chosen (Horus+ HD Video Otoscope, JedMed[R], St. Louis, MO, USA). Images are initially recorded on SD cards in the field and subsequently transferred to hard discs at the research office.

3. A portable, rechargeable battery-operated tympanometry and distortion product otoacoustic emission (DPOAE) device (Path Sentiero[R], Path Medical, Germering, Germany, model SOD07) was selected. While the device has many functionalities, only a limited number were required for the study. The 226Hz frequency tympanogram issued at a pressure range of -600 to +400 daPa. The tympanometer outputs for each ear include: a tracing viewable by the tester as well as physical measurements of ear canal volume (ml), peak pressure (daPa), peak compliance (ml) and tympanic width (daPa). DPOAE is used as a screening tool and measured at 2, 3, and 4 kHz for each ear. Although detailed results are available through the device, only the basic results with outputs as “pass” or “refer” are to be used since DPOAE is only being used as a screening tool for hearing loss.

4. A portable, rechargeable battery-operated screening audiometry system (HearScreen[R], HearX, Pretoria, South Africa) has been chosen coupled with a smartphone (Samsung Galaxy 12) and an ISO calibrated noise-cancelling headphone (Sennheiser HD280-pro Circumural Headphone [0.5 – 8kHz]). The device uses an automated testing protocol to test pure tone frequencies at 0.5, 1, 2, 3, 4, 6 and 8 kHz for each ear at sound intensity levels of 20 to 70 dB HL. The device then generates a pass or fail interpretation based on published best practice guidelines. Subjects that fail the screening audiogram are

For peer review only - http://bmjopen.bmj.com/site/about/guidelines.xhtml

For peer review only

referred for a diagnostic audiogram at Bohol Hearing Center. All HearX data are stored on remote servers and can be accessed by the research team.

E. Training of local research personnel

The training of local Filipino nurses by a U.S. otolaryngology/audiologist team has been described previously.[12] Briefly, 4 nurses were trained didactically on the topic of OM and the basics of audiology and its relationship to OM clinically. Then they were given hands-on training in otoscopy, cerumen removal, video-otoscopy, and all of the audiologic equipment to perform tympanometry, DPOAE, and screening audiograms. The training session culminated in the nursing team obtaining video-otoscopic, audiologic, and clinical data from 47 subjects under direct supervision of the U.S. otolaryngologist/audiologist team.

F. Protocols for data collection, storage and transfer protocols

Research data collection by the local research team include: 1) case report forms; 2) video-otoscopy and audiologic testing; and 3) miscellaneous data.

1. Case report form (CRF)

Each research subject has an original paper CRF which contains patient identifiers and clinical information entered by the research personnel following a parental interview with ear-specific past medical history (i.e. ear infection, discharge, TM perforation, and hearing loss). CRFs collected during the week are uploaded by the research nurse into a REDCap database hosted at the University of Colorado Denver School of Medicine, Aurora, Colorado, USA.

For peer review only - http://bmjopen.bmj.com/site/about/guidelines.xhtml

2. Video-otoscopy and audiologic testing

Subjects whose TMs are devoid of cerumen or whose cerumen can be cleaned by the research nurses undergo video-otoscopy and audiologic testing. All subjects whose cerumen cannot be cleaned adequately are given a bottle of mineral oil and a medicine dropper and advised to use 5 drops twice a day for 7 days and asked to return for another assessment. However, subjects that fail to have adequate cerumen removal after the subsequent visit or had active otorrhea are referred to the local otolaryngologist for further management

The research nurses record the 2 ears of a research subject in a regimented sequence for ease of review by the APPs at a subsequent time (right ear still photo, left ear still photo, right ear video, and left ear video). SD cards are downloaded at the end of each weekday and images stored on 2 hard disks (original and backup disks). Data is transferred to the US by external drives

Audiologic testing data are processed in the following manner. The 4 discreet outputs produced by the tympanometer are used to determine tympanogram types. An overlay “rectangle” onto the tympanometer tracing is used by the manufacturer to include most “normal” tympanograms within these confines as defined by Jerger[13] to assist with diagnosis. The local research team uses this real-time judgement call for a “pass” or “fail” depending if the peak of the tracing is within the “normal” ranges as dictated by the “rectangle.” DPOAE results are also automatically determined by the device as either “pass” or “refer.” Hearing screening done on subjects using the HearScreen

For peer review only - http://bmjopen.bmj.com/site/about/guidelines.xhtml

device also classifies subjects as "pass" or "fail" by the device. All subjects who fail the tympanogram, DPOAE, or HearScreen are referred to Bohol Hearing Center for diagnostic audiometry. All raw tympanogram, automated DPOAE, automated HearScreen, and raw HearX data are entered by the research supervisor to the REDCap database on a weekly basis.

3. Miscellaneous data

All audiologic data from Bohol Hearing Center, as well as all otologic diagnoses made by the island otolaryngologist, are also entered into the REDCap database by the research supervisor on an as-needed basis.

G. Cataloging of tympanometric data

The tympanometric data for each ear consisting of ear canal volume (ml), peak pressure (daPa), peak compliance (ml), and tympanic width (daPa) are sorted based on the classic classification established by Jerger: A, B, C, A_s, and A_d types. As such, a portion of data would result in tympanometric parameters landing outside of the confines for each type defined by Jerger.[13] Since the research team viewed it imperative that each video-otoscopy be accompanied by a tympanogram type to aid in video-otoscopy interpretation, an *ad hoc* methodology had to be developed to capture the "non-classifiable" tympanograms. The study otolaryngologist and a senior audiologist independently will read all "non-classifiable" tympanograms, and discordant classifications will be adjudicated by a second senior audiologist. The majority vote will confirm the classification type.

For peer review only - http://bmjopen.bmj.com/site/about/guidelines.xhtml

H. Video-otoscopy data and diagnosis outcome classifications

Video-otoscopy analysis leading to disease classification takes into account all otoscopic abnormalities associated with acute OM, chronic non-suppurative OM, and CSOM, as well as a non-OM diagnoses (e.g. otitis externa) summarized in Table 1. APPs document findings rather than immediately arrive at a diagnosis. Each video-otoscopic image is associated with a tympanogram type as to simulate pneumatic otoscopy, a proxy for the gold standard to diagnose middle-ear effusion.[14,15] The gold standard for the presence of middle-ear fluid is detected only by myringotomy; however, this being impossible, the proxy methodology used here is based on the decisions made by the senior pediatric otolaryngologist with over 30 years of performing otologic procedures for complications, from OM including tympanoplasties and mastoidectomies. Given it would be impractical and impossible for the pediatric otolaryngologist to view thousands of video-otoscopies, experienced otoscopists (APPs) have been trained and validated in identifying otoscopic abnormalities listed in Table 1.

All video-otoscopies that are deemed as poor quality by the APP are reviewed by the otolaryngologist of the study and would remained classified as poor quality or into one or more of the following TM findings.

Video-otoscopic findings for each ear will then merged with the 4 tympanometric output parameters and clinical past medical history to arrive at discreet diagnoses (Figure 1). Four broad diagnostic categories with definitions include normal, mild, moderate, and severe ear disease. If two ears of a subject have disparate levels of middle-ear disease, the subject would be classified as having the more serious form of ear disease for the purpose of analysis.

For peer review only - http://bmjopen.bmj.com/site/about/guidelines.xhtml

1. Normal

No evidence of ear disease as determined by no disease-related video-otoscopic findings regardless of tympanometric abnormal states and clinical past medical history of ear disease.

2. Mild Ear Disease

- Acute otitis media (AOM) – The presence of at least two of the following three otoscopic signs: 1) white, yellow, amber, or blue TM (i.e., abnormal color), 2) opacification not due to scarring, 3) tympanometry peak compliance <0.2 ml or tympanometric width > 200 daPa on tympanometry; or bubbles or air-fluid interfaces; and one of the following three signs/symptoms: a) ear pain, b) redness of the TM, c) fullness or bulging of the TM or acute purulent otorrhea not due to otitis externa.[16-18]

- Otitis media with effusion (OME) – The presence of at least two of the following three otoscopic signs: 1) white, yellow, amber, or blue TM (i.e., abnormal color), 2) opacification not due to scarring, 3) tympanometry peak compliance <0.2 ml or tympanometric width > 200 daPa from tympanogram; or bubbles or air-fluid interfaces; and the absence of the following four signs/symptoms: a) ear pain, b) redness of the TM, c) fullness or bulging of the TM, or d) acute purulent otorrhea not due to otitis externa.[16-18]

- Healed Perforation – Otherwise normal TM with a thinned-out area of TM.

For peer review only - http://bmjopen.bmj.com/site/about/guidelines.xhtml

- Myringosclerosis – a hardening of the ear drum as calcium deposits form on the ear drum and middle ear; peak compliance on tympanogram > 5.0 ml.

3. Moderate Ear Disease

- Dry Perforation – perforation of the TM without otorrhea or ear canal volume ≥2 ml on tympanogram.

- Adhesive otitis media – Intact TM but retraction or retraction pocket seen.

4. Severe Ear Disease

- Chronic Suppurative Otitis Media (CSOM), active – perforation of the TM with otorrhea duration of more than 2 weeks; or TM with retraction pocket unable to see deepest part, with granulation tissue, squamous debris, or otorrhea.

I. **Protocol to deal with ears with incomplete data set**

It is expected some subjects or ears would not have a complete data set. Reasons would include poor quality collection techniques, uploading error and the subjects not following through with seeing the island otolaryngologist or completing the audiogram at Bohol Hearing Center.

When the case report form indicates otorrhea and no other data is available/discernable, these ears are classified as CSOM. When the case report form indicates no otorrhea or ear pain and only the tympanogram is available, the ears are classified either as normal or OME based on type A or types B/C, respectively. In ears without tympanograms, ear disease classification is solely based on video-otoscopy. When the case report form notes no ear pain, tympanogram is

For peer review only

For peer review only - http://bmjopen.bmj.com/site/about/guidelines.xhtml

missing and video-otoscopy is missing/undiscernible, these ears are deemed “missing data” and will not be included as part of the final analysis.

J. Training of U.S. advanced practice providers

Three outpatient APPs from the Department of Otolaryngology at Children’s Hospital Colorado with varying length of tenure and training in otoscopy ($\geq$7 years of clinical experience) were trained in reading video-otoscopic images using didactic in-person training methods to identify the 14 possible otoscopic signs described in Table 1.

K. Patient and Public Involvement

No patient and public involvement took place in the design, or conduct, or reporting, or dissemination plans of the research.

L. Statistical Analysis Methodology

Demographic attributes and potential risk factors derived from the Parent Questionnaire will be analyzed to determine which ones should be included in the multivariate analyses. A *P*-value $\leq$ 0.15 will be used to select the ‘significant’ variables. Odds ratios will be computed for the dichotomous categorical variables, using a Cochran’s Mantel-Haenszel statistic to compute the *P*-value. Continuous variables and categorical variables with more than two categories will be analyzed using Student’s *t*-test or Wilcoxon Rank-Sum test. A Rank-Sum test will be used when the assumptions required for the *t*-test are not met. When there are significant differences in ear disease classification between the vaccine and placebo group, then a multivariate logistic

For peer review only

regression model will be derived to identify the vaccine efficacy for the 11PCV vaccine, and significant effects for the covariables.

DISCUSSION

The methodology discussed in this manuscript will be utilized to analyze a potential cohort of 12,000 children previously enrolled in a pneumococcal vaccine trial in Bohol, Philippines and will now be assessed 12 to 18 years later for the vaccine's effects on otoscopic and audiologic endpoints. It is unique in several aspects: size of the cohort, clinical endpoints, and complexity of data analysis.

The sample sizes of previously published randomized controlled pneumococcal conjugate vaccine trials have been clearly summarized by a recently published Cochrane Review.[19] Our cohort of over 12,000 children to be assessed likely would be lowered since attrition might occur due to inability to locate the families due to relocation or immigration after 15 to 18 years, as well as parental refusal to participate. Regardless, the biggest pneumococcal vaccine trials were published by Black et al in 2000[20-22] and Tregnaghi et al in 2014[23] involving 37,868 and 24,000 subjects, respectively. Black's study was conducted in the U.S., and acute OM was the only ear-related outcome.[21] The Tregnaghi study was done in Central and South American countries, but only 7,214 children from Panama were included in the OM portion of the study.[23] Prymula et al in 2006 published an acute OM trial using an 11-valent pneumococcal vaccine conjugated to *Hemophilus influenzae*-derived protein D trial in 4,968 subjects.[24] This trial was conducted in the Czech Republic and Slovakia, and tympanocentesis and pneumococcal subtyping were done. A recent open label trial of maternal immunization with a 23 valent

For peer review only - http://bmjopen.bmj.com/site/about/guidelines.xhtml

polysaccharide pneumococcal vaccine in 227 Indigenous mothers in the Northern Territories, Australia, showed no impact on OM related infant outcomes at 7 months of age.[25]

The clinical outcomes for the randomized controlled trials have included the reduction of pneumococcal OM, recurrent OM, OM visits, and tympanostomy tube insertions.[20-24,26] None of these randomized controlled trials were focused on the long-term sequalae of OM in terms of changes in the TM as evaluated by otoscopy and changes in hearing as evaluated by audiometry. While prevention of disease is compelling for the use of the pneumococcal vaccine, preventing of long-term sequalae are just as important. Trials conducted in the Indigenous populations in Australia that examined long-term sequelae occurred after licensure of pneumococcal vaccines in Australia and so followed before/after designs.[27-30] Thus, our study will be among the largest randomized controlled pneumococcal vaccine trials for OM and the only one to study the long-term effects on OM sequelae 15 - 18 years after the initial trial.

The complexity of the methodology dealing with data lies at several levels. First, it requires the raw data collected by the local research team be uniform and robust. Despite a local research supervisor ensuring good quality data were recorded and uploaded, a certain degree of data scrubbing will be required on the U.S. side. Tympanometric and audiologic data are codified based on published definitions of classifications. An algorithm has also been developed to derive OM diagnostic endpoints from all video-otoscopic images that was reliant on the raw images and tympanogram types.

CONCLUSIONS

For peer review only - http://bmjopen.bmj.com/site/about/guidelines.xhtml

The overall construct of the methodology may serve as a platform for conducting large scale OM research in LMIC, particularly, if the objectives include garnering otoscopic and audiologic determinates and assessment of chronic and long-term OM complications.

ETHICS AND DISSEMINATION

A. Ethics

The “Do No Harm” precept has been inculcated in the study. Despite the healthcare discrepancy that exists between the U.S. and Bohol, Philippines, accommodations have been made in the methodology to refer the research subjects to local healthcare professionals. The local field workers will be instructed to refer subjects to Bohol Hearing Center for failed OAEs, tympanometry, and/or screening audiometry and to the local otolaryngologist for ear pathology (inability to remove cerumen after instillation of mineral oil from a previous visit, otorrhea and suspicion for cholesteatoma).

The study was approved by the Research Institute of Tropical Medicine, Alabang, Manila, Philippines, and the Institution Review Board and the Colorado Multiple Institutional Review Board of the University of Colorado School of Medicine, Aurora, Colorado, USA.

Study data are collected and managed using REDCap electronic data capture tools hosted at University of Colorado. REDCap (Research Electronic Data Capture) is a secure, web-based application designed to support data capture for research studies, providing: 1) an intuitive interface for validated data entry; 2) audit trails for tracking data manipulation and export procedures; 3) automated export procedures for seamless data downloads to common statistical packages; and 4) procedures for importing data from external sources. Data are

For peer review only - http://bmjopen.bmj.com/site/about/guidelines.xhtml

stored, managed and processed within the system. Data are exported as SAS files to secure servers on the university of Colorado and Children's Hospital Colorado systems, where data will be analyzed.

B. Dissemination Research data is privately held. Research results will be made available to children and their caregivers with abnormal audiologic outcomes, the funders and other researchers. After publication anonymized and curated data, will be made available to researchers following guidelines of the Bill and Melinda Gates foundation. Data will be published in open access journals only.

For peer review only

For peer review only - http://bmjopen.bmj.com/site/about/guidelines.xhtml

Authors' Contributions

Conception and acquisition of funding- Simoes

Epidemiologic and Field Design – Simoes, Tallo, Lucero, De Jesus, Bautista, Sanvictores

Otoralyngologic Study design: Chan, Uhler, Simoes, Carosone-Link, Uhler

Audiologic study design: Uhler, Chan, Simoes, Carosone-Link, Uhler

Analysis – Simoes, Chan, Carosone-Link, Uhler

Redcap Database creation – Carosone-Link

Drafting and revision – all authors

For peer review only

Funding Statement

This study was supported by the Bill & Melinda Gates Foundation (grant number OPP1142570) and Colorado CTSA Grants UL1TR002535, KL2TR002534, and TL1TR002533 and UL1 RR025780. The contents of this report are solely the responsibility of the authors and do not necessarily represent the official views of their institutions or organizations or of the sponsors. The funders did not participate in any aspect of the study, including study-conduct, data collection, analyses of the data or the write-up of the manuscript. The corresponding authors confirm that they had full access to all the data in the study and had final responsibility for the decision to submit for publication.

Competing interests

The authors declare that they have no competing interests.

Acknowledgements

The authors, on behalf of study team, would like to thank the other nurses who were the subjects of training: Nino Carlo Salutan, Leslie Salutan, Marelie Dagupan and Shobelle Anunciado; the staff of Research Institute of Tropical Medicine, Manila Philippines for their dedication to their patients, including our trial participants; Deborah Hayes, PhD who was instrumental in developing the audiology protocols.

For peer review only

REFERENCES:

1. Acuin J. Chronic suppurative otitis media : burden of illness and management options. Geneva: World Health Organization; 2004.
2. Adebola SO, Ayodele SO, Oyelakin OA, Babarinde JA, Adebola OE. Pre-school hearing screening: profile of children from Ogbomoso, Nigeria. *Int J Pediatr Otorhinolaryngol* 2013; **77**(12): 1987-91.
3. Adhikari P, Joshi S, Baral D, Kharel B. Chronic suppurative otitis media in urban private school children of Nepal. *Braz J Otorhinolaryngol* 2009; **75**(5): 669-72.
4. Feniman MR, Souza AG, Jorge JC, Lauris JR. Otoscopic and tympanometric findings in infants with cleft lip and palate. *Braz J Otorhinolaryngol* 2008; **74**(2): 248-52.
5. Mahomed-Asmail F, Swanepoel de W, Eikelboom RH. Hearing loss in urban South African school children (grade 1 to 3). *Int J Pediatr Otorhinolaryngol* 2016; **84**: 27-31.
6. Okur E, Yildirim I, Akif Kilic M, Guzelsoy S. Prevalence of otitis media with effusion among primary school children in Kahramanmaras, in Turkey. *Int J Pediatr Otorhinolaryngol* 2004; **68**(5): 557-62.
7. Smith AF, Ianacone DC, Ensink RJH, Melaku A, Casselbrant ML, Isaacson G. Prevalence of hearing-loss among HAART-treated children in the Horn of Africa. *Int J Pediatr Otorhinolaryngol* 2017; **98**: 166-70.
8. Zakzouk SM, AbdulJawad KA. Point prevalence of type B tympanogram in children. *Saudi Med J* 2002; **23**(6): 708-10.
9. Anggraeni R, Hartanto WW, Djelantik B, et al. Otitis media in indonesian urban and rural school children. *Pediatr Infect Dis J* 2014; **33**(10): 1010-5.
10. Simoes EA, Kiio F, Carosone-Link PJ, Ndegwa SN, Ayugi J, Macharia IM. Otitis Media and Its Sequelae in Kenyan Schoolchildren. *Journal of the Pediatric Infectious Diseases Society* 2015.
11. Lucero MG, Nohynek H, Williams G, et al. Efficacy of an 11-valent pneumococcal conjugate vaccine against radiologically confirmed pneumonia among children less than 2 years of age in the Philippines: a randomized, double-blind, placebo-controlled trial. *Pediatr Infect Dis J* 2009; **28**(6): 455-62.
12. Chan KH, Dreith S, Uhler KM, et al. Large-scale otoscopic and audiometric population assessment: A pilot study. *Int J Pediatr Otorhinolaryngol* 2018; **117**: 148-52.
13. Jerger J. Clinical experience with impedance audiometry. *Arch Otolaryngol* 1970; **92**(4): 311-24.
14. Nozza RJ, Bluestone CD, Kardatzke D, Bachman R. Identification of middle ear effusion by aural acoustic admittance and otoscopy. *Ear Hear* 1994; **15**(4): 310-23.
15. Kaleida PH, Fireman P. Diagnostic assessment of otitis media. *Clin Allergy Immunol* 2000; **15**: 247-62.
16. Diagnosis and management of acute otitis media. *Pediatrics* 2004; **113**(5): 1451-65.
17. Morris PS, Leach AJ, Halpin S, et al. An overview of acute otitis media in Australian Aboriginal children living in remote communities. *Vaccine* 2007; **25**(13): 2389-93.
18. Pellman H. All otitis media is not created equal. *J Pediatr* 1999; **135**(2 Pt 1): 264.

For peer review only - http://bmjopen.bmj.com/site/about/guidelines.xhtml

19. Fortanier AC, Venekamp RP, Boonacker CW, et al. Pneumococcal conjugate vaccines for preventing acute otitis media in children. *The Cochrane database of systematic reviews* 2019; **5**: Cd001480.
20. Black SB, Shinefield HR, Ling S, et al. Effectiveness of heptavalent pneumococcal conjugate vaccine in children younger than five years of age for prevention of pneumonia. *Pediatr Infect Dis J* 2002; **21**(9): 810-5.
21. Fireman B, Black SB, Shinefield HR, Lee J, Lewis E, Ray P. Impact of the pneumococcal conjugate vaccine on otitis media. *Pediatr Infect Dis J* 2003; **22**(1): 10-6.
22. Black S, France EK, Isaacman D, et al. Surveillance for invasive pneumococcal disease during 2000-2005 in a population of children who received 7-valent pneumococcal conjugate vaccine. *Pediatr Infect Dis J* 2007; **26**(9): 771-7.
23. Tregnaghi MW, Saez-Llorens X, Lopez P, et al. Efficacy of pneumococcal nontypable Haemophilus influenzae protein D conjugate vaccine (PHiD-CV) in young Latin American children: A double-blind randomized controlled trial. *PLoS Med* 2014; **11**(6): e1001657.
24. Prymula R, Peeters P, Chrobok V, et al. Pneumococcal capsular polysaccharides conjugated to protein D for prevention of acute otitis media caused by both Streptococcus pneumoniae and non-typable Haemophilus influenzae: a randomised double-blind efficacy study. *Lancet* 2006; **367**(9512): 740-8.
25. Binks MJ, Moberley SA, Balloch A, et al. PneuMum: Impact from a randomised controlled trial of maternal 23-valent pneumococcal polysaccharide vaccination on middle ear disease amongst Indigenous infants, Northern Territory, Australia. *Vaccine* 2015; **33**(48): 6579-87.
26. Pettigrew MM, Alderson MR, Bakaletz LO, et al. Panel 6: Vaccines. *Otolaryngol Head Neck Surg* 2017; **156**(4_suppl): S76-s87
27. Mackenzie GA, Carapetis JR, Leach AJ, Morris PS. Pneumococcal vaccination and otitis media in Australian Aboriginal infants: comparison of two birth cohorts before and after introduction of vaccination. *BMC Pediatr* 2009; **9**: 14.
28. Leach AJ, Wigger C, Andrews R, Chatfield M, Smith-Vaughan H, Morris PS. Otitis media in children vaccinated during consecutive 7-valent or 10-valent pneumococcal conjugate vaccination schedules. *BMC Pediatr* 2014; **14**: 200.
29. Leach AJ, Mulholland EK, Santosham M, et al. Pneumococcal conjugate vaccines PREVenar13 and SynflorIX in sequence or alone in high-risk Indigenous infants (PREV-IX_COMBO): protocol of a randomised controlled trial. *BMJ open* 2015; **5**(1): e007247.
30. Leach AJ, Wigger C, Beissbarth J, et al. General health, otitis media, nasopharyngeal carriage and middle ear microbiology in Northern Territory Aboriginal children vaccinated during consecutive periods of 10-valent or 13-valent pneumococcal conjugate vaccines. *Int J Pediatr Otorhinolaryngol* 2016; **86**: 224-32.

For peer review only

LEGENDS OF TABLE AND FIGURE

Table. List of video-otoscopic finds depicting physical findings associated with otitis media

Figure. Schema in classifying tympanometric types based on tympanometer outputs (accordance with Jerger classification)

For peer review only

TABLE

- Redness of TM
- Opacification not due to scarring (dullness; thickened; webbing)
- Bubbles or air-fluid interfaces or fluid
- Abnormal TM color: white, yellow, amber, or blue
- Fullness or bulging of TM
- Thinned out area of TM or healed perforation seen in 360°
- Tympanosclerosis/Scarring
- Perforation SMALL (<25% of TM)
- Perforation LARGE (≥25% of TM)
- Retraction/Retraction pocket/generally thinned out but not healed TM
- Retraction/ pocket bottom not seen
- Otorrhea not due to otitis externa (moisture)
- Granulation tissue or squamous debris
- Otitis externa

For peer review only

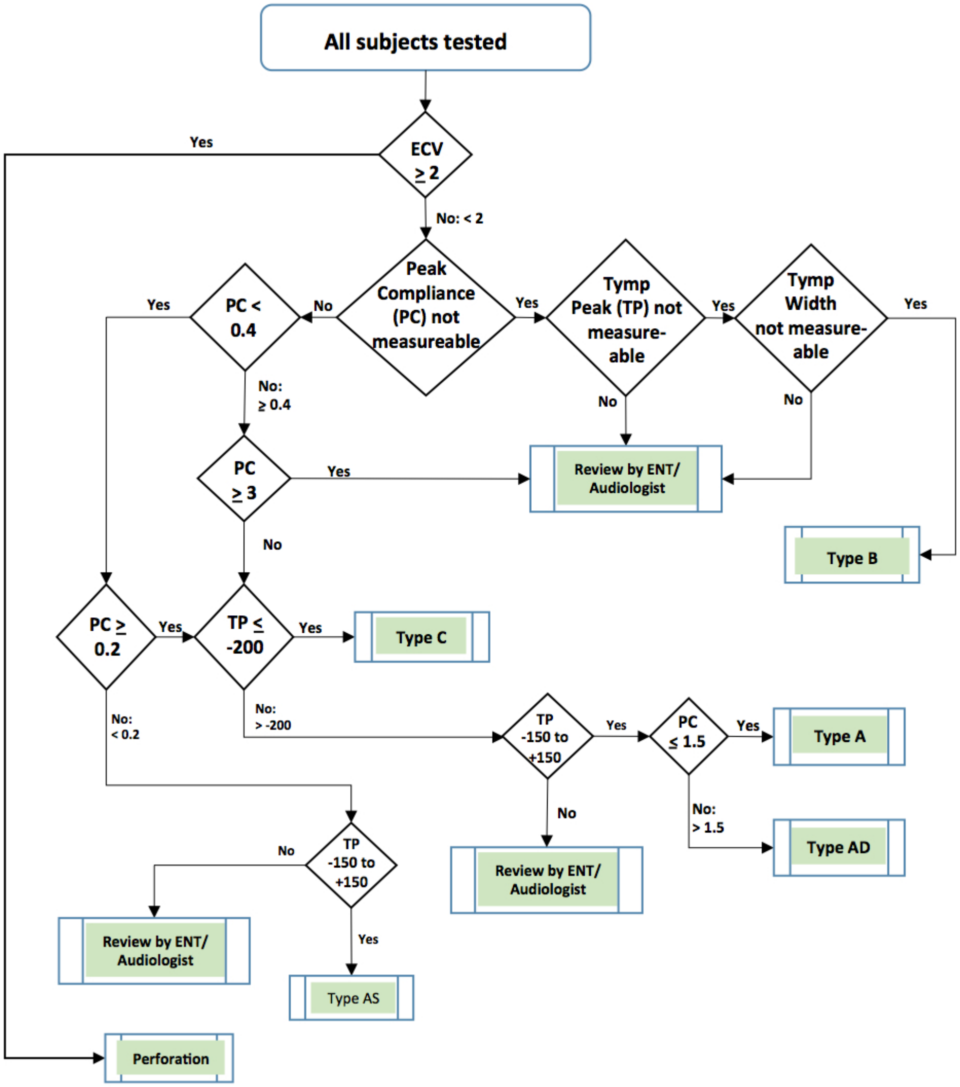

Figure 1. Schema in classifying tympanometric types based on tympanometer outputs (accordance with Jerger classification)

82x90mm (300 x 300 DPI)
